# Impacts of demographic and laboratory parameters on key hematological indices in an adult population of southern Taiwan: A cohort study

**DOI:** 10.1371/journal.pone.0201708

**Published:** 2018-08-02

**Authors:** Ming-Chung Wang, Cih-En Huang, Meng-Hung Lin, Yao-Hsu Yang, Chang-Hsien Lu, Ping-Tsung Chen, Yu-Ying Wu, Hsing-Yi Tsou, Chia-Chen Hsu, Chih-Cheng Chen

**Affiliations:** 1 Division of Hematology and Oncology, Department of Medicine, Chang Gung Memorial Hospital, Kaohsiung, Taiwan; 2 Division of Hematology and Oncology, Department of Medicine, Chang Gung Memorial Hospital, Chiayi, Taiwan; 3 Graduate Institute of Clinical Medical Sciences, College of Medicine, Chang Gung University, Taoyuan, Taiwan; 4 Center of Excellence for Chang Gung Research Datalink, Chang Gung Memorial Hospital, Chiayi, Taiwan; 5 Institute of Occupational Medicine and Industrial Hygiene, National Taiwan University College of Public Health, Taipei, Taiwan; 6 School of Traditional Chinese Medicine, College of Medicine, Chang Gung University, Taoyuan, Taiwan; 7 Department of Traditional Chinese Medicine, Chang Gung Memorial Hospital, Chiayi, Taiwan; National Institutes of Health, National institute of Diabetes and Digestive and Kidney Diseases, UNITED STATES

## Abstract

Studies in Caucasians have shown that values of hematological indices could be affected by a wide variety of factors. However, parallel work in other ethnical populations, particularly from the Asia-Pacific region, is lacking. Therefore, we designed this study to explore the association between clinical/laboratory parameters and hemogram levels. Adult individuals who came to our hospital for health exams were screened. Information on demographics and laboratory profiles was obtained. We analyzed the impacts of these parameters on the variation of hemogram. Overall, 26,497 adults were included in the current analysis after excluding those with abnormal hemogram. Multivariate regression analysis showed increasing age and male gender negatively affected the number of platelets, whereas a higher serum apolipoprotein B level was associated with an elevated platelet count. Gender and serum albumin level were the major determinants of variation in hemoglobin level. A modestly increased white cell count was seen in men as well as individuals with elevated apolipoprotein B levels, but it was inversely correlated with changes in age and serum albumin levels. Conversely, some variables, although statistically significantly associated with the hematological indices, only provided a trivial explanation for the heterogeneity observed. We further established predictive models for the approximate estimation of hematological indices in healthy adults. Our data indicate that age, gender, and serum levels of apolipoprotein B and albumin affect hematological indices in various ways. We also demonstrate that variation in hemogram could be successfully predicted by a number of clinical and laboratory parameters.

## Introduction

Complete blood counts are one of the most commonly ordered laboratory blood tests. Several key hematological indices, particularly the white blood cell (WBC) count and platelet count, have been associated with atherosclerotic diseases and cardiovascular deaths [1, 2]. Variation in the hemogram could, therefore, significantly contribute to meaningful clinical consequences. Epidemiologic studies suggest that age and gender are the major determinants in the heterogeneity of hematological indices [3–6], whereas more recent evidence have demonstrated that lipid profiles also contribute to the change in these parameters [7, 8]. Furthermore, the disparate genetic backgrounds among ethnic groups may also play a role, as a genome-wide meta-analysis has identified 12 loci being associated with the variation in several hematological parameters [9]. However, our understanding of the impacts of clinical and biochemical parameters on key hematological indices, particularly their reciprocal interaction at a population level, is limited.

Through a feedback program sponsored by the largest petrochemical corporation in Taiwan, a significant proportion of residents from two rural villages in southern Taiwan underwent an annual health examination in our hospital during the last 7 years. The results of these exams were kept in the Chang Gung Research Datalink of Chang Gung Memorial Hospital, Taiwan. We took advantage of this and retrieved data on age, gender, nutritional level, lipid profile, and hepatitis B or C serological testing results to study their association with several key hematological indices We analyzed the individual impact of each parameter on the changes in hemogram levels.

## Methods

### Study population

With the establishment of the Sixth Naphtha Cracker Complex in southern Taiwan, the Formosa Plastics Group started a feedback program for residents of adjacent townships in 2010. The program allowed inhabitants from Mailiao and Taishi villages of Yunlin county, Taiwan, to come to our hospital for free annual health exams. The epidemiological background as well as the laboratory data of those examined individuals was retained in the Chang Gung Research Datalink of Chang Gung Memorial Hospital, Taiwan. Upon the approval of the current study by the IRB of Chang-Gung Memorial Hospital, we retrieved demographic information including age and gender, the results of hemogram and serological testing for hepatitis B (HBV) and C (HCV), and data on the levels of total cholesterol (TC), triglyceride (TG), apolipoprotein B (Apo-B), albumin, and hepatic transaminases of the participating individuals, for the current analysis. Only adults who were 18 years or older at the time of examination were included. All the clinical data, after assigning a coding number for each subject, were encrypted without identifiable personal information and provided to the investigators of the current study team.

### Hematological and biochemical measurements

For blood cell analysis, a Sysmex XE-5000 hematology analyzer (Sysmex; Kope, Japan) was used [10]. Measurement of all biochemical parameters was performed using a Hitachi Labospect 008 clinical analyzer (Hitachi, Ltd.; Tokyo, Japan). The TC was assayed based on coupled enzymatic reactions, followed by spectrophotometric detection [11]. The Apo-B level was quantified by a turbidimetric immunoassay [12]. The bromocresol green dye-binding method was used to measure the serum albumin level [13]. Serum triglyceride level measurement was based on an initial lipolysis to yield glycerol and free fatty acids, followed by a sequence of three coupled enzymatic reactions, catalyzed by glycerol kinase, L-α-glycerophosphate oxidase and peroxidase enzymatic reactions, to form a red quinoneimine dye, which was determined spectrophotometrically [14]. Serum levels of alanine aminotransferase and aspartate aminotransferase were quantified by the enzymatic rate method [15]. Detection of hepatitis B surface antigen (HBsAg) [16] and anti-HCV antibody [17] was performed by chemiluminescent microparticle immunoassay using an Analytics E170 analyzer (Roche Diagnostics; Indiana, USA).

### Selection of study cohort

The aim of the current study was to evaluate the impacts of clinical and biochemical parameters on key hematological indices in healthy adults. Therefore, we only selected clinically assessed healthy or normal individuals for the current analysis. The purpose was to exclude subjects with possible hematological diseases and those with cytopenia resulting from non-hematological conditions (including, but not limited to, medication, toxic chemicals, viral infections, chronic inflammation, and immune disorders). We reasoned that inclusion of these individuals might inadvertently lead to biased estimation of potentially confounding factors on the variation in hemogram. The preset ranges were as follows: 3.5–11.0 × 10^9^/L for the total WBC, 0–3.0 × 10^9^/L for the absolute lymphocyte count (ALC), an absolute neutrophil count (ANC) of 1.8 x 10^9^/L or greater, a hemoglobin (Hb) level of 12–16 g/dL for females and 13–16.5 g/dL for males, 80–100 fL for the mean corpuscular volume (MCV) of red blood cells, and 100–450 × 10^9^/L for the platelet count. The reference range for the WBC count was based on that measured in normal individuals at our institute. The selection of upper limits of the Hb and platelet counts was based on the thresholds used to define polycythemia vera and essential thrombocythemia in the 2016 revised World Health Organization (WHO) classification of myeloid neoplasms [18]. The ANC level and lower limit of platelet count (100 × 10^9^/L) followed the WHO thresholds, which define cytopenia for the diagnosis of myelodysplastic syndrome (MDS) in the 2016 revised classification of myeloid neoplasms [18, 19]. For both genders, the lower Hb thresholds selected in this study were based on the recently recommended cytopenia levels for aiding the diagnosis of MDS by a panel of experts [20].

In order to avoid the confounding effects of abnormal cytopenia (particularly, thrombocytopenia) secondary to HBV- or HCV-related chronic hepatitis might exert on the variation of normal hemogram [21, 22], we excluded individuals who were positive for HBsAg or anti-HCV antibody tests and had elevated serum aminotransferase levels. However, hepatitis B or C carriers with normal liver function tests were allowed for the current analysis.

### Statistical analysis

We used linear regression models, based on the method of generalized estimating equations (GEE), to assess the association between hematological indices and various factors, accounting for multiple blood test records in the same subject. Univariate analysis was first performed to evaluate the potential effects of an individual factor on hemogram, whereas multivariate linear regression was employed to appraise the impacts of key confounding factors on the variation of these hematological indices. All statistical analyses were set at a two-sided *p*-value of 0.05 significance level and were performed with SAS version 9.4 (SAS Institute, Inc., Cary, NC, USA).

## Results

In all, more than 70,000 blood testing records from a total of 26,497 adult individuals were included in the current analysis. The study cohort was rather evenly distributed in both genders, as it contained 13,101 (49.4%) adult females (Table 1). More than half of the enrolled subjects were 44-years of age or younger (n = 14,194; 53.6%), whereas about one-eighth (n = 3,213; 12.1%) of the whole population were elderly (≥ 65 years) individuals. As we excluded chronic hepatitis B or C patients in our current study, HBV and HCV carriers accounted for only 5.6 and 3.7% of the entire cohort, respectively. With the small number of hepatitis carriers enrolled, we did not further analyze the impact of hepatitis carrier status on hematological indices. Table 2 shows the distribution of the whole data in percentile ranges, which contained the number of records and the results of various biological, serological, and hematological parameters.

**Table 1:** Baseline characteristics of the enrolled individuals.

Variables	No.	%
**Total**	26497	
**Gender**		
** Female**	13101	49.4
** Male**	13396	50.6
**Age (yrs)**		
** 18–44**	14194	53.6
** 45–64**	9090	34.3
** ≥65**	3213	12.1
**HBV**		
** Yes**	1482	5.6
** No**	25015	94.4
**HCV**		
** Yes**	991	3.7
** No**	25506	96.3

**Table 2:** The distribution of the whole data in percentile ranges.

Variables	N*	Min.^	1st^∞^	5th^∞^	50th^∞^	95th^∞^	99th^∞^	Max.^
**TC (mg/dL)** ^#^	70504	14	117	136	186	248	284	1270
**TG (mg/dL)** ^#^	69262	18	36	45	98	270	476	9375
**Apo-B (mg/dL)** ^#^	37836	10	44	56	89	133	157	272
**Albumin (g/dL)** ^#^	54798	0.3	3.3	4.0	4.5	5.0	5.2	6.8
**PLT (1000/μL)** ^#^	66144	100	117	149	234	342	397	450
**HGB (g/dL)** ^#^	55953	12	12.5	12.9	14.6	16.3	16.5	16.5
**MCV (fL)** ^#^	59620	80	80.9	83.1	89.3	96	98.7	100
**WBC (μL)** ^#^	69331	3500	3700	4150	6250	9500	10600	11000
**ANC (μL)** ^#^	59101	1801	1912	2185	3611	6346	7778	10379
**ALC (μL)** ^#^	56061	14	729	1103	1919	2772	2948	3000

*N: the number of records.

^Min.: minimal values; Max.: maximal values

^∞^1^st^–99^th^: 1^st^ represents data at the 1^st^ percentile range of distribution, and 99^th^ represents those at the 99^th^ percentile range.

^#^Abbreviations: TC: total cholesterol; TG: triglyceride; Apo-B: Apolipoprotein B; PLT: platelet count; HGB: hemoglobin; MCV: mean corpuscular volume; WBC: white blood cell count; ANC: absolute neutrophil count; ALC: absolute lymphocyte count.

### Effects of an individual factor on hemogram by univariate analysis

Tables 3 to 8 demonstrated the effects of an individual factor on the variation of several key hematological indices by univariate analysis. Gender was the sole dichotomous variable, whereas continuous variables contained age, as well as serum levels of TC, TG, Apo-B and albumin. For statistical purposes, we further divided these continuous variables into categorical classes, with the estimated changes based on a per 10-year increment for age, per 10-mg/dL increment for TC, TG, and Apo-B, and a per 1-g/dL increment for albumin. When looking at the impacts of certain parameters on specific hematological indices, three factors were considered of ultimate importance. First, a single parameter had to be statistically significant, with a two-sided *p*-value ≤ 0.05. Second, the degree of impacts of the parameters on hematological indices mattered. For example, the gender factor affected both platelet count and MCV, with *p* < 0.001 in both instances (Table 3). However, unlike the significant gap between platelet counts in both genders, the MCV difference between men and women was only 0.61 fL, which was essentially irrelevant in clinical settings. Third and most critically, the fluctuation ranges of the parameters could magnify differences between hematological indices to discrepant degree. Taking the Apo-B serum levels for example, the platelet counts were increased by 1.63 × 10^9^/L for every 10 mg/dL increment of Apo-B in univariate analysis (Table 7). With the respective levels of serum Apo-B between the 5^th^ and 95^th^ percentiles of the total population being 56 and 133 mg/dL (Table 2), an individual with a serum Apo-B level of 133 mg/dL would have 13.04 × 10^9^/L (or eight times 1.63 × 10^9^/L) more platelets than an individual with a serum Apo-B level of 56 mg/dL. On the contrary, the platelet count was more pronouncedly influenced by serum albumin level, as the platelet count was increased by 9.78 × 10^9^/L for every 1 g/dL increment of albumin in univariate analysis (Table 8). However, due to the small variation in serum albumin levels in this cohort (1 g/dL difference between individuals at the 5^th^ and 95^th^ percentiles, see Table 2), we considered that there were probably little differences between the impacts of serum levels of albumin and Apo-B on the platelet counts in our study cohort.

**Table 3:** Gender effects on hematological indices (female as the reference group).

Indices	Estimate	95% CI	p-value
**PLT (1000/μL)** ^#^	-23.02	(-24.35 to -21.68)	<0.001
**HGB (g/dL)** ^#^	1.48	(1.46 to 1.50)	<0.001
**MCV (fL)** ^#^	0.61	(0.52 to 0.71)	<0.001
**WBC (μL)** ^#^	466	(432 to 499)	<0.001
**ANC (μL)** ^#^	193	(167 to 220)	<0.001
**ALC (μL)** ^#^	72	(61 to 83)	<0.001

^#^Abbreviations: PLT: platelet count; HGB: hemoglobin; MCV: mean corpuscular volume; WBC: white blood cell count; ANC: absolute neutrophil count; ALC: absolute lymphocyte count.

**Table 4:** Age effects on hematological indices (per 10-year increment).

Indices	Estimate	95% CI	p-value
**PLT (1000/μL)** ^#^	-10.17	(-10.55 to -9.80)	<0.001
**HGB (g/dL)** ^#^	-0.09	(-0.10 to -0.08)	<0.001
**MCV (fL)** ^#^	0.46	(0.44 to 0.49)	<0.001
**WBC (μL)** ^#^	-100	(-110 to -90)	<0.001
**ANC (μL)** ^#^	-13.38	(-21.82 to -4.95)	0.002
**ALC (μL)** ^#^	-62.04	(-65.4 to -58.64)	<0.001

^#^Abbreviations: PLT: platelet count; HGB: hemoglobin; MCV: mean corpuscular volume; WBC: white blood cell count; ANC: absolute neutrophil count; ALC: absolute lymphocyte count.

**Table 5:** Total cholesterol effects on hematological indices (per 10-mg/dL increment).

Indices	Estimate	95% CI	p-value
**PLT (1000/μL)** ^#^	1.20	(1.04 to 1.35)	<0.001
**HGB (g/dL)** ^#^	0.03	(0.03 to 0.04)	<0.001
**MCV (fL)** ^#^	0.03	(0.02 to 0.04)	<0.001
**WBC (μL)** ^#^	19.34	(15.32 to 23.35)	<0.001
**ANC (μL)** ^#^	-1.24	(-4.69 to 2.21)	0.482
**ALC (μL)** ^#^	12.72	(11.37 to 14.06)	<0.001

^#^Abbreviations: PLT: platelet count; HGB: hemoglobin; MCV: mean corpuscular volume; WBC: white blood cell count; ANC: absolute neutrophil count; ALC: absolute lymphocyte count.

**Table 6:** Triglyceride effects on hematological indices (per 10-mg/dL increment).

Indices	Estimate	95% CI	p-value
**PLT (1000/μL)** ^#^	-0.011	(-0.044 to 0.021)	0.498
**HGB (g/dL)** ^#^	0.012	(0.007 to 0.017)	<0.001
**MCV (fL)** ^#^	0.001	(-0.002 to 0.004)	0.714
**WBC (μL)** ^#^	19.68	(15.96 to 23.39)	<0.001
**ANC (μL)** ^#^	11.39	(9.18 to 13.60)	<0.001
**ALC (μL)** ^#^	4.99	(3.76 to 6.22)	<0.001

^#^Abbreviations: PLT: platelet count; HGB: hemoglobin; MCV: mean corpuscular volume; WBC: white blood cell count; ANC: absolute neutrophil count; ALC: absolute lymphocyte count.

**Table 7:** Apolipoprotein-B effects on hematological indices (per 10-mg/dL increment).

Indices	Estimate	95% CI	p-value
**PLT (1000/μL)** ^#^	1.63	(1.14 to 1.86)	<0.001
**HGB (g/dL)** ^#^	0.07	(0.07 to 0.08)	<0.001
**MCV (fL)** ^#^	-0.08	(-0.09 to -0.06)	<0.001
**WBC (μL)** ^#^	68	(62 to 76)	<0.001
**ANC (μL)** ^#^	29	(23 to 35)	<0.001
**ALC (μL)** ^#^	24	(21 to 26)	<0.001

^#^Abbreviations: PLT: platelet count; HGB: hemoglobin; MCV: mean corpuscular volume; WBC: white blood cell count; ANC: absolute neutrophil count; ALC: absolute lymphocyte count.

**Table 8:** Albumin effects on hematological indices (per 1-g/dL increment).

Indices	Estimate	95% CI	p-value
**PLT (1000/μL)** ^#^	9.78	(8.15 to 11.42)	<0.001
**HGB (g/dL)** ^#^	0.84	(0.81 to 0.87)	<0.001
**MCV (fL)** ^#^	-0.36	(-0.46 to -0.26)	<0.001
**WBC (μL)** ^#^	-1.90	(-54.64 to 50.85)	0.944
**ANC (μL)** ^#^	-242	(-284 to -201)	<0.001
**ALC (μL)** ^#^	239	(224 to 253)	<0.001

^#^Abbreviations: PLT: platelet count; HGB: hemoglobin; MCV: mean corpuscular volume; WBC: white blood cell count; ANC: absolute neutrophil count; ALC: absolute lymphocyte count.

Compared to women, men had a higher WBC count and Hb level but fewer platelets (Table 3). Increasing age adversely affected almost all key hematological parameters, particularly WBC, ALC, and platelets, yet its impacts on the levels of Hb and ANC were less pronounced (Table 4). For the lipid profiles, it seemed that both TC and TG levels minimally influenced the hemogram variation (Tables 5 and 6), whereas an increasing Apo-B level had a modestly positive effect on WBC count (an increment of 0.068 × 10^9^/L per 10 mg/dL rise in Apo-B; Table 7). Individuals with higher albumin levels had increased Hb levels (Table 8). However, as above-mentioned, due to the small variation in serum albumin levels in this cohort, its effects on other hematological indices were considered minimal (Table 8).

In order to test the independent influence of various clinical and biochemical parameters, we employed a multivariate regression model to examine their association with the hemogram variation (Table 9). Given the good correlation between the levels of TC and Apo-B in our study cohort (correlation coefficient *r* = 0.82211, *p* < 0.0001, Pearson’s correlation; data not shown), we did not incorporate TC in our multivariate analysis.

**Table 9:** Multivariate regression model for prediction of hematological indices based on meaningful clinical/biological parameters.

	PLT (1000/μL) ^#^		HGB (g/dL) ^#^		MCV (fL) ^#^		WBC (μL) ^#^		ALC (μL) ^#^	
Variable	Estimate	*P*-value	Estimate	*P*-value	Estimate	*P*-value	Estimate	*P*-value	Estimate	*P*-value
**Intercept**	275.993	<0.001	10.118	<0.001	85.905	<0.001	6619.576	<0.001	1522.008	<0.001
Gender (male)*	-26.174	<0.001	1.396	<0.001	0.517	<0.001	392.220	<0.001	46.165	<0.001
**Age (years)**^†^	-1.178	<0.001	-0.002	<0.001	0.064	<0.001	-15.510	<0.001	-6.437	<0.001
**TG (mg/dL)**^†^	0.007	0.003	0.001	<0.001			1.500	<0.001	0.353	<0.001
**Apo-B (mg/dL)**^†^	0.301	<0.001	0.005	<0.001	-0.015	<0.001	6.781	<0.001	2.776	<0.001
**Albumin (g/dL)**^†^	1.968	0.046	0.714	<0.001	0.232	<0.001	-113.924	0.001	97.055	<0.001

* Female as the reference groups.

^†^The estimate is changes for age per 1 year, TG per 1 mg/dL, Apo-B per 1 mg/dL, and Albumin per 1 g/dL increment, respectively.

^#^Abbreviations: TG: triglyceride; Apo-B: Apolipoprotein B; PLT: platelet count; HGB: hemoglobin; MCV: mean corpuscular volume; WBC: white blood cell count; ANC: absolute neutrophil count; ALC: absolute lymphocyte count.

### Variation in WBC count and ALC

The average WBC count was 0.392 × 10^9^/L higher in men than women. Regarding the effects of continuous variables on the WBC count, age was the most influential factor (a decrement of 15.51/μL in WBC count for every 1-year increment in age), which was followed by serum Apo-B level (an increment of 6.781/μL in WBC for every 1 mg/dL increment in Apo-B level). The increase in serum albumin levels seemed to significantly decrease the WBC count, yet considering that the variation in serum albumin levels in this cohort was small, the impact of albumin on WBC count was considerably less than age and Apo-B.

With regards to ALC, age as well as serum albumin and Apo-B levels were probably the three most influential factors, although their effects on ALC were modest at best. As shown in Table 9, ALC increased by 2.776/μL for every 1 mg/dL increment in Apo-B level and decreased by 6.437/μL for every 1-year increment in age. For every 1 g/dL increment, serum albumin level increased ALC by 97.055/μL. The effects of gender and serum levels of TG on ALC, although statistically significant, were minimal.

### Impacts of clinical/biochemical parameters on red cell indices

It is well known that there is an apparent discrepancy in the Hb levels between both genders, and the difference was 1.396 g/dL in our study cohort. Nutritional status also significantly impacted the Hb level. The Hb level increased 0.71 g/dL for every 1 g/dL increment in albumin level. Conversely, the influence of age and serum levels of TG and Apo-B on Hb level was negligible (Table 9).

It seemed that the red cells increased in size with increasing age in our cohort. The variation in the MCV of red cells was 0.064 fL for every 1-year difference in age. Conversely, the impacts of other parameters on MCV were inconsequential (Table 9).

### Association between platelet counts and key variables

In our study, men had, on average, 26.174 × 10^9^/L fewer platelets than women. Age also significantly affected the number of platelets. The platelet counts decreased by 1.178 × 10^9^/L for every 1-year increment in age. From a practical perspective, there would be a difference of around 85 × 10^9^/L in platelet counts between a 20-year-old adult female and a 70-year-old healthy male. This showed that the combinative effects of age and gender on platelet counts could be immense. The serum Apo-B level also exerted a modest influence on the platelet counts because the number of platelets increased by 0.301 × 10^9^/L for every 1 mg/dL increment in Apo-B levels. Lastly, there was an inessential rise in the platelet counts with the increase in both serum TG and albumin levels in the whole population.

### Models for prediction of hematological indices

To establish a useful model for the prediction of hematological indices, we excluded statistically insignificant parameters and repeated the multivariate regression analysis. The results were demonstrated in Table 9, with an intercept added for each key hematological index. Through this model, expected values or estimated ranges of hemogram can be calculated by interpolating data on demographic as well as biochemical parameters.

## Discussion

With significant variation in hemogram across various ethnic populations being repeatedly reported in the literature [3, 6, 23, 24], large-scale data from different geographic regions are urgently needed. Herein, we present the interactive association between clinical/biochemical parameters and hematological indices from a cohort of more than 26,000 Taiwanese, enrolling by far the largest population of adult healthy individuals from the Asia-Pacific region for such a study. Furthermore, the inclusion of all potentially relevant information together in the study allows dissecting the separate independent impacts on hemogram, immensely adding to the strength and integrity of our investigation.

Age, gender, and the serum Apo-B level are the three most important factors affecting WBC counts in our study. Our finding of a gender effect on WBC echoed a previous study in a Chinese Han population [25], although in contrast to the current study, the authors did not identify any link between age and WBC count. A second study similarly showed a higher WBC in men than in women, but the effect in that study was apparent only in those who were 50 years or older [26]. With regards to the serum Apo-B level, there have been reports showing correlations between WBC count and metabolic syndrome, as higher WBC counts are linked to adverse lipid profiles associated with this condition [27, 28]. We demonstrated a positive correlative effect between the serum Apo-B level and WBC count, although the correlation was only modest (an increment of 6.781/μL in WBC for every 1 mg/dL increment in Apo-B levels).

Through the mechanisms of chronic menstrual blood loss in females and a higher androgen level in males, it has been well documented that men have a significantly higher Hb concentration than women [26]. Therefore, it is not surprising that there is a 1.4 g/dL difference in Hb levels between both genders in our study. We also observed that the serum albumin level was the second most influential factor on the Hb variation, as the level of Hb increased by 0.71 g/dL for every 1 g/dL increment in albumin levels. A higher level of albumin is probably associated with better nutritional status and, consequently, a lower chance of deficiencies in iron, vitamin B_12_ or folate [29]. From the univariate analysis, age adversely impacted the Hb level (-0.09 g/dL for every 10-year increase in age). However, when factors, such as levels of TG, Apo-B, and albumin, were considered together in multivariate analysis, the impact of age on Hb decreased, whereas the influence of the albumin level stood out (Table 9). There have been few, albeit contradictory, reports on the link between red cell abundance and serum lipid profiles [30, 31]. The prospective National Health and Nutrition Examination Survey (NHANES) in the United States showed a positive connection between the levels of non–high-density lipoprotein cholesterol and Hb [8]. However, like our study, the effect of serum lipid levels on the red cell abundance in that cohort was, although statistically significant, minimal.

The variation in the size of red cells in our study population was small, except for the influence of age. Age had a modest impact on MCV, as the size of red cells enlarged with increasing age. This finding is consistent with the work by Hoffmann *et al*., which also reveals an age-related increase in MCV in a cohort of more than 8,000 individuals [32].

Not surprisingly, age and gender were the two most influential factors on the platelet count variations in our cohort, a finding that was consistently reported in previous studies [5, 23, 24, 26, 33]. The exact mechanism behind a gradual decrease in platelet counts with an increasing age is not immediately clear through an observational study. It could be the result of a survival advantage in individuals with lower platelet counts, or simply indicative of a reduced hematopoietic stem cell reserve that comes with aging [5, 33]. Conversely, it is more comprehensible that female adults have more platelets than men. The total body iron storage is generally lower in women because of menstruation, and iron depletion is a well-known factor to stimulate platelet production [34, 35]. Furthermore, a higher serum estrogen level in women might also play a role in the increased platelet counts, as studies have revealed that estrogen favorably benefits platelet production [4, 36].

Our study also shows a modestly positive effect of the serum Apo-B level on the number of platelets, a result in line with the prospective NHANES study which discloses an association between the levels of non-high density lipoprotein-cholesterol and platelet counts [8]. Thrombocytes have been demonstrated to exhibit a high capacity for diffusional exchange of cholesterol with plasma [37, 38], and *in vitro* incubation of platelets with cholesterol-enriched lipid dispersions could lead to cholesterol incorporation into the cellular membrane, which would thereby regulate the cellular population. The roles of lipid content on potentiating thrombopoiesis are further supported by evidence showing a cholesterol-induced increase in stromal cell-derived factor-1 and thrombopoietin receptor [39, 40]. Moreover, reduced serum cholesterol levels could represent a status of malnutrition. This might result in thrombocytopenia through the mechanism of folate or vitamin B_12_ deficiency.

Genetic variation might also play a role in the heterogeneity of platelet count, an important factor that we were not able to appraise in such a study of retrospective nature. However, previous reports have demonstrated that genetic and ethnic factors are responsible for the fluctuation in the number of platelets [3–6, 33]. Also, a meta-analysis has identified 12 genetic loci associated with platelet count variation [9]. Analysis of the genetic background with available DNA samples, in conjunction with all the demographic and biochemical parameters, shall help delineate their collaborative effects on the number of platelets in the future.

So, what does this study offer? Our data can be considered invaluable by serving as a guide to establish the reference ranges of hematological indices in healthy Taiwanese adult individuals. Second, while the considerable impacts of clinical and biochemical parameters on hemogram identified in our study are of uncertain clinical significance, increasing age, leukocytosis, thrombocytosis, and increased Apo-B levels, are all risk factors for thromboembolic diseases. These variables may synergize in promoting cardiovascular diseases. For instance, with the active roles of leukocytes and thrombocytes in pro-inflammatory and pro-coagulant activities [41–43], and considering the positive effects of the serum Apo-B level on WBC and platelet counts in our cohort, the impacts of Apo-B on the risk of cardiovascular events, besides its participation in the atherosclerotic process [44], are probably higher than believed. Therefore, it is justifiable to simultaneously incorporate hemogram into traditional cardiovascular risk factors, such as diabetes, hypertension, smoking, serum Apo-B levels and age, to assess an individual’s probability of developing coronary artery disease or cerebral vascular attack. This shall lead to better risk-stratified management in the prevention of cardiovascular diseases.

Another potential utility of our findings is for the detection of clonal hematopoiesis of indeterminate potential (CHIP) in previously unsuspected individuals, or for the prognostication of those who harbor clonal hematopoiesis but are otherwise free from cytopenias. Initially identified in two large groups of people without known hematological diseases [45, 46], CHIP is seen in more than 10% of those who are 70 years or older. Clonal hematopoiesis has been defined as a premalignant state based on the elevated risk of clonal progression and subsequent development of hematological malignancies, with the hazard ratios of the risk of progression being 11.1–12.9 [45–47]. In our prediction model, we can roughly estimate expected values of hematological indices in healthy people. If the differences between the detected and expected values are significant, we can further investigate if such individuals are more likely to harbor CHIP. Alternatively, in those with clonal hematopoiesis and seemingly normal hemogram whose hematological indices are considerably lower than expected, we can monitor them more frequently to ascertain if they are more likely to progress to overt neoplasms. If so, it probably means those individuals have clonal cytopenia of undetermined significance (CCUS, a condition with an inherently higher risk of progression than CHIP [47]) instead of CHIP because their seemingly normal hematological indices are actually abnormal based on their own standard. Detection of CHIP and differentiation between CHIP and CCUS has implications both for refined prognostication and for potential interventions that could avert progression or modify disease risk. Conversely, a similar inference can be used to identify individuals at risk of being in a pre-myeloproliferative neoplasm state, even if the hemogram appears normal. Future, longitudinal, cohort studies of these aspects would be mandatory, and the results of our work lay an important foundation for serving as a reference guide and assist rationally designed researches aimed at identifying when to detect CHIP and how to monitor CHIP-carrying individuals.

Despite the valuable information provided by the current analysis, the study is limited in several aspects. There was inevitably significant selection bias in this study, since it only included people from Southern Taiwan who participated in the health screening process sponsored by the petrochemical company. This limits the generalizability of the results. Second, we excluded individuals whose hemogram fell outside the pre-defined ranges. This might lead to bias due to exclusion of some adults who were otherwise healthy but had hematological indices in assumed “abnormal” levels. However, we thought this would be more appropriate than inadvertently including patients with significant diseases into our study cohort and leading to flawed results or an incorrect conclusion.

In summary, our data illustrate the variability of key hematological indices associated with the heterogeneity of healthy adult individuals’ characteristics. Not surprisingly, demographic data including age and gender are the two most influential factors on the hemogram variation. Among the biochemical parameters, serum Apo-B levels modestly explain the heterogeneity of platelet count and WBC count, whereas serum albumin levels are positively correlated with higher Hb concentrations. We also establish predictive models for approximate estimation of hematological indices in adult healthy individuals. With our predictive models and the availability of DNA samples stored in our biological research bank, large-scale screening of hematological neoplasm-associated mutations and serial follow-up of mutant allele burden as well as hemogram will help better understanding of the natural course and molecular basis of clonal hematopoiesis in the near future.
